# Computational Insights into Root Canal Treatment: A Survey of Selected Methods in Imaging, Segmentation, Morphological Analysis, and Clinical Management

**DOI:** 10.3390/dj13120579

**Published:** 2025-12-03

**Authors:** Jianning Li, Kerstin Bitter, Anh Duc Nguyen, Hagay Shemesh, Paul Zaslansky, Stefan Zachow

**Affiliations:** 1 Zuse Institute Berlin, Takustrasse 7, 14195 Berlin, Germany; zachow@zib.de; 2 Department of Operative Dentistry and Periodontology, Medical Faculty, Martin-Luther-University Halle-Wittenberg, Magdeburger Strasse 16, 06112 Halle (Saale), Germany; kerstin.bitter@uk-halle.de (K.B.); anh.nguyen@uk-halle.de (A.D.N.); 3 Department of Endodontology, Academic Centre for Dentistry Amsterdam, Gustav Mahlerlaan 3004, 1081 LA Amsterdam, The Netherlands; h.shemesh@acta.nl; 4 Department for Operative, Preventive and Pediatric Dentistry, Charité—Universitätsmedizin Berlin, Aßmannshauser Str. 4-6, 14197 Berlin, Germany; paul.zaslansky@charite.de; 5 Department of Oral and Maxillofacial Surgery, Charité—Universitätsmedizin Berlin, Augustenburger Platz 1, 13353 Berlin, Germany

**Keywords:** root canal therapy, endodontics, dental imaging, micro-computed tomography, image segmentation, computer-assisted diagnosis

## Abstract

**Background/Objectives:** Root canal treatment (RCT) is a common dental procedure performed to preserve teeth by removing infected or at-risk pulp tissue caused by caries, trauma, or other pulpal conditions. A successful outcome, among others, depends on accurate identification of the root canal anatomy, planning a suitable therapeutic strategy, and ensuring a bacteria-tight root canal filling. Despite advances in dental techniques, there remains limited integration of computational methods to support key stages of treatment. This review aims to provide a comprehensive overview of computational methods applied throughout the full workflow of RCT, examining their potential to support clinical decision-making, improve treatment planning and outcome assessment, and help bridge the interdisciplinary gap between dentistry and computational research. **Methods:** A comprehensive literature review was conducted to identify and analyze computational methods applied to different stages of RCT, including root canal segmentation, morphological analysis, treatment planning, quality evaluation, follow-up, and prognosis prediction. In addition, a taxonomy based on application was developed to categorize these methods based on their function within the treatment process. Insights from the authors’ own research experience were also incorporated to highlight implementation challenges and practical considerations. **Results:** The review identified a wide range of computational methods aimed at enhancing the consistency and efficiency of RCT. Key findings include the use of advanced image processing for segmentation, image analysis for diagnosis and treatment planning, machine learning for morphological classification, and predictive modeling for outcome estimation. While some methods demonstrate high sensitivity and specificity in diagnostic and planning tasks, many remain in experimental stages and lack clinical integration. There is also a noticeable absence of advanced computational techniques for micro-computed tomography and morphological analysis. **Conclusions:** Computational methods offer significant potential to improve decision-making and outcomes in RCT. However, greater focus on clinical translation and development of cross-modality methodology is needed. The proposed taxonomy provides a structured framework for organizing existing methods and identifying future research directions tailored to specific phases of treatment. This review serves as a resource for both dental professionals, computer scientists and researchers seeking to bridge the gap between clinical practice and computational innovation.

## 1. Introduction

**Figure 1:**
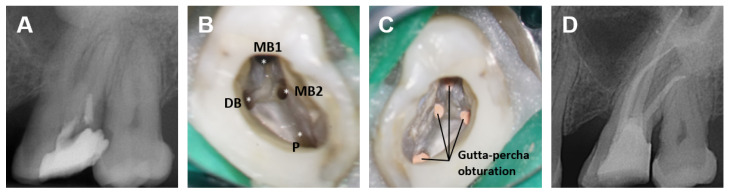
Illustration of a typical RCT procedure. (**A**): Pre-operative X-ray. (**B**): Instrumented root canals (marked with asterisks, including Mesiobuccal canal 1 (MB1), Mesiobuccal canal 2 (MB2), Distobuccal canal (DB) and Palatal canal (P)) after final irrigation using Sodium hypochlorite (NaOCl) and Ethylenediaminetetraacetic acid (EDTA). (**C**): Gutta-percha-obturated root canals. (**D**): Post-operative X-ray.

**Figure 2:**
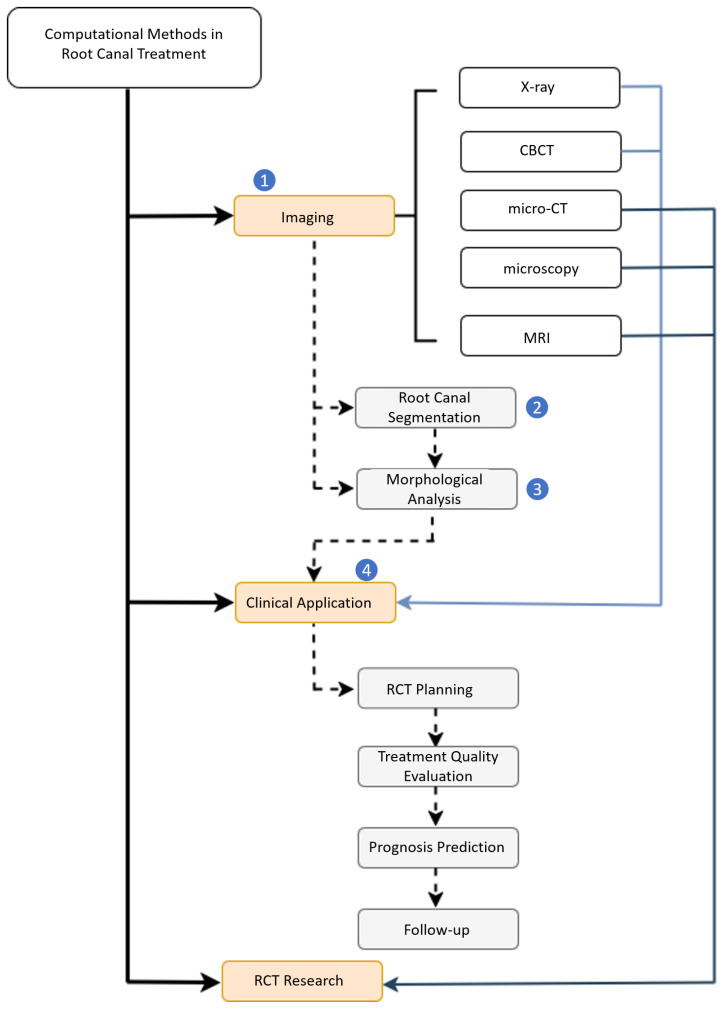
Taxonomy for the review of computational methods in root canal treatment. The reviewed methods are categorized into different stages, from root canal segmentation and morphological analysis to key RCT procedures. Additionally, methods designed for RCT research and clinical applications are clearly distinguished with respect to the dental imaging modalities involved. Note that, the evaluation of prognosis and restorability is included within the treatment planning phase of RCT. Numbers 1–4 in the taxonomy denote different RCT stages.

**Figure 3:**
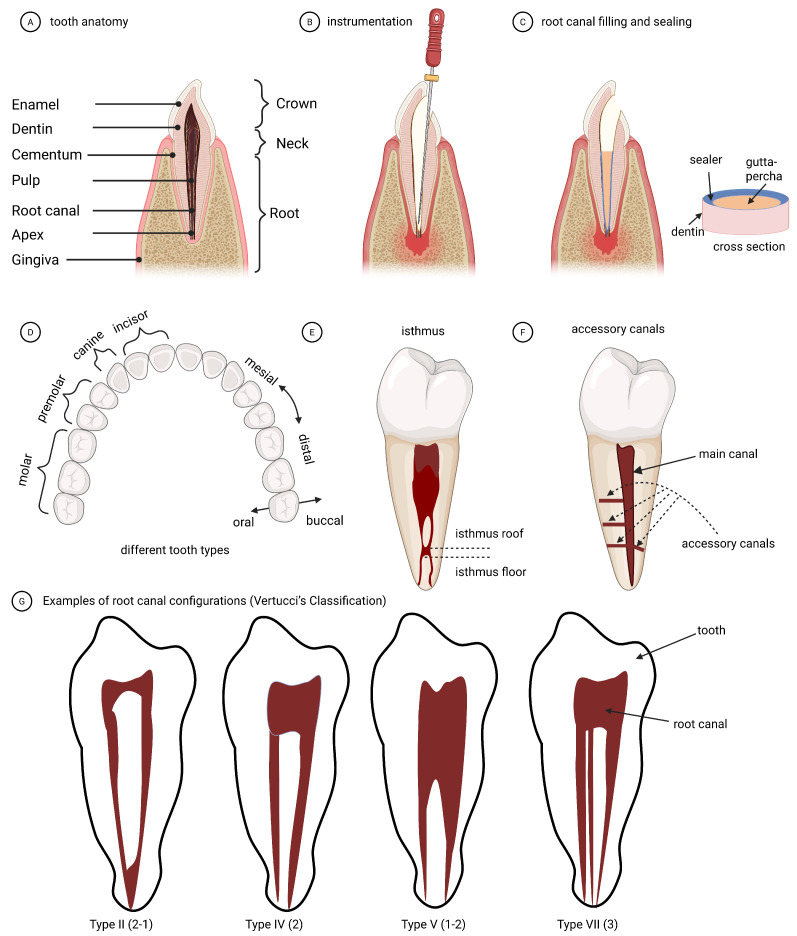
(**A**) Illustrations of tooth internal anatomy. (**B**) RCT instrumentation. (**C**) Root canal filling and sealing. (**D**–**F**) Illustration of dental terms related to RCT, including different types of teeth, isthmus and accessory canals. Both structures increase anatomical complexity and challenge cleaning and obturation. (**G**) Examples of different root canal configurations (RCC). This figure was created using BioRender.

Root canal treatment (RCT) is a dental procedure that involves the removal of pulp tissues, or pulp tissues at risk of future pathology, from the root canal, followed by cleaning, shaping, filling, and sealing the canals. It is the standard treatment option for severely decayed or traumatized teeth. RCT is indicated for teeth affected by irreversible pulpitis, pulp necrosis, trauma, cracked teeth, or extensive restorative needs. Figure 1 depicts the general clinical workflow of an RCT procedure, including (1) radiological diagnosis and pre-operative X-ray (Figure 1A); (2) removing pulp tissue, disinfecting using proper irrigating solutions (Figure 1B), and shaping the root canals via instrumentation (Figure 1B); (3) root canal filling and sealing using gutta-percha (Figure 1C); and (4) post-operative radiographic assessment using X-rays (Figure 1D). The procedure’s success largely depends on thorough disinfection, root canal filling, and tight coronal sealing of the tooth (Figure 1C), which are influenced by the endodontist’s expertise and the morphological complexity of the root canal system [1]. Ideal sealing creates a bacteria-tight interface between the dentin and filling material, while the entire root canal is filled without gaps or voids [2]. The root canal system can be highly intricate and variable, exhibiting multiple branches and irregular shapes that vary based on the tooth type (incisor, canine, premolar, molar) and individual anatomical differences. Such morphological complexity makes the process of disinfecting, filling and sealing the canals during RCT more challenging, often leading to less predictable treatment outcome and prognosis [3].

Imaging techniques are essential for understanding these complexities, allowing clinicians to assess treatment outcomes based on the procedures used, the materials applied, and the unique structure of each root canal. Leveraging well-established dental imaging techniques such as X-ray and cone beam computed tomography (CBCT) for clinical diagnosis, along with microscopy and micro-computed tomography (micro-CT) for research, various computational methods have been developed to analyze or support different stages of the RCT process. These methods range from traditional image processing and machine learning algorithms to advanced deep learning-based approaches, aiding in tasks such as root canal segmentation and morphological analysis, RCT planning, treatment quality assessment, prognosis prediction, and follow-up evaluations. This review provides an overview of these methods for general readers, dentists, and computer scientists who are interested in endodontics or dentistry, in general, breaking the ‘language barrier’ between the dentistry and computer science fields. By doing so, it ensures that both communities can understand the content without the need for specialized background knowledge. The review also identifies under-explored areas and proposes directions for future research, ultimately offering computational insights to support clinical RCT management.

### 1.1. Taxonomy

We present a taxonomy, illustrated in Figure 2, to cohesively categorize the computational methods, which forms the foundation for the content and structure of this review. The taxonomy starts with various dental imaging techniques including X-ray, CBCT, micro-CT, microscopy and magnetic resonance imaging (MRI). In this context, we differentiate between dental imaging techniques used in clinical practice (e.g., X-ray, CBCT) and those primarily employed in clinical research, due to their ex vivo, destructive, or still experimental nature (e.g., micro-CT, microscopy, MRI). The gray boxes in the taxonomy diagram represent the six main categories of the computational methods reviewed, namely, root canal segmentation, morphological analysis of the root canal system, RCT planning, treatment quality evaluation, as well as prognosis prediction and follow-ups. The dashed arrow in the diagram represents the typical workflow of the computational methods, which includes the following steps: (1) acquisition of dental images based on specific clinical or research needs; (2) image segmentation of the region of interest (ROI) of the root canal system, including dentin, pulp, as well as sealer and filling materials in treated teeth; (3) morphological analysis of the root canal system to assess variations in complexity; and (4) clinical application of those computational insights and derivation of measures for an improved RCT management. We also identified the computational methods that translate knowledge acquired from research-focused imaging to clinical routine imaging, bridging the gap between dental research and clinical practice [4,5,6].

### 1.2. Dental Terminologies

**Figure 4:**
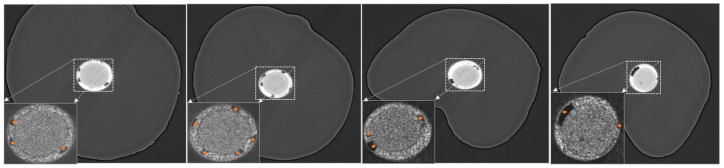
The micro-structures of RCT defects visualized using Phase-Contrast Enhanced (PCE) micro-CT, with the zoomed-in root canals shown in the bottom-left corner of each image. Dark areas inside the sealer area/layer in the root canal indicate pores/defects (marked with asterics).

**Figure 5:**
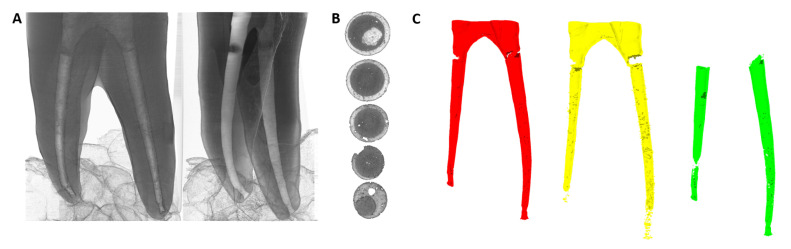
(**A**) PCE micro-CT Volume rendering of the root canals from a treated premolar. (**B**) Cross-sectional visualizations. (**C**) Segmentation of the root canal (red), sealer (green) and root filling (gutta-percha, yellow) from the PCE micro-CT.

Table 1 presents the dental terms necessary for understanding the dental context of the review. Each dental term is explained in simple language to ensure easy understanding for those without a dental background, eliminating the need for additional resources. Furthermore, the majority of these dental terms are illustrated in Figure 3, Figure 4 and Figure 5.

### 1.3. Manuscript Outline

Section 1 and Section 2 provide the essential background knowledge to understand the context of RCT, including dental imaging, root canal anatomies, and RCT in clinical routine, which ensures a smooth transition to the technical discussion of the computational methods. In Section 3, the computational methods applied to various stages of RCT are presented, compared and analyzed to draw computational insights that can be translated to clinical practice in RCT management, as well as obtaining a general understanding of possible complications and resulting failures of RCT. Section 4 provides a comprehensive presentation of these findings and insights, along with practical recommendations derived from our research experience, for the development and implementation of computational methods for supporting RCT.

### 1.4. Search Strategy and Scope of Review

To find relevant papers, we performed a search exclusively on Google Scholar using keywords such as root canal, root canal treatment, endodontics together with additional keywords such as ‘segmentation’, ‘classification’, ‘morphology’, ‘planning’, ‘prognosis’, ‘treatment quality’, ‘deep learning’. Google Scholar was selected due to its broad coverage across both clinical dental publications and computer science venues, including conference proceedings (e.g., MICCAI, CVPR, ICCV) where many state-of-the-art computational methods are published but may not yet appear in traditional medical databases. The search was led primarily by the first author J. Li, who has a biomedical engineering and computer science background. Note that our review does not aim to exhaustively capture all published works, but to select representative studies aligned with each category of our proposed taxonomy (Figure 2), covering computational methods relevant to key stages of RCT management. The taxonomy is designed in consultation with the dental professionals among the co-authors. Additionally, studies involving large language models (LLMs) [7] or dental record analytics [8], as well as computational approaches for tooth or crown modeling outside the context of RCT, were excluded. To further refine the scope of this review, we do not cover computational methods aimed at diagnosing or detecting conditions for RCT [9], as our scope centers on computational support within the treatment workflow.

## 2. Dental Imaging in Root Canal Treatment

This section starts by introducing the basics of the RCT process (Section 2.1), followed by a discussion of the common dental imaging in clinical practice (Section 2.2) and endodontic research (Section 2.3). It is recommended that readers, particularly those outside the field of endodontics, familiarize themselves with the dental terms summarized in Table 1 before proceeding further. The internal tooth anatomies, root canal morphologies, and the RCT process, including instrumentation, filling, and sealing, are depicted in Figure 3 to facilitate a visual and intuitive understanding of these terms mentioned throughout this review. The characteristics of different dental imaging modalities in the context of RCT are summarized in Table 2.

### 2.1. Root Canal Treatment

Before RCT, the root canal system exists in its native or potentially inflammed or infected state, with irregular shapes that vary between tooth types and individuals. The pulp still contains nerves and blood vessels, and the inner dentine remains intact. During RCT, the caries-affected dentin and other infected organic materials are removed, and an access opening is prepared through the existing crown to reach the pulp chamber and root canals. Instrumentation (e.g., performed by endodontic files) is used to clean and shape the root canal system, followed by disinfection. This process, also called root canal preparation [10], reshapes the canal into a more uniform form (Figure 4 and Figure 5), leaving an empty canal space ready for filling and sealing. Improper instrumentation, influenced by variations in the operator’s skill, such as over-instrumentation and excessive pressure, can damage the inner dentine [11,12,13,14]. Additionally, failure to thoroughly clean the root canal apex or other narrow canal branches, such as lateral canals, can also lead to treatment failure, due to insufficient bacterial elimination. After the canals have been prepared and filled with gutta-percha and a root canal sealer, which should create a bacteria-tight seal against the root canal dentin wall and ensure complete filling of the root canals, ideally free from defects such as gaps, voids and pores, preventing bacterial reinfection. Refer to Figure 4 for examples of RCT defects. The morphological complexity of the root canal system (Figure 3), e.g., the high curvature, the existence of lateral canals, isthmus and subtle branches, directly influences the instrumentation, disinfection, filling and sealing processes, and is one of the most important aspects to consider for treatment planning, post-treatment quality evaluation, prognosis prediction and follow-ups [15,16,17,18,19].

### 2.2. Dental Imaging in RCT Clinical Routine

X-ray and CBCT are common imaging tools in clinical RCT practice for planning, post-treatment evaluation, and follow-ups. As shown in Figure 1, X-rays can be used for assessing basic root canal anatomy, such as the location and number of canals, their length and curvature, and the apex, although the number of canals can sometimes be difficult to determine due to over-projection. X-rays are also employed for immediate post-treatment evaluation to assess the quality of the procedure as well as for routine follow-ups. CBCT is selectively used for more complex cases with irregular canal morphologies, providing detailed 3D imaging of the root canal system, bone structures, or root fractures [20] and enabling a more precise evaluation of the filling and sealing quality.

### 2.3. Dental Imaging in RCT Research and Education

Microscopy, micro-CT and phase-contrast enhanced (PCE) micro-CT provide ultra-high-resolution imaging of the root canal system and capture the fine micro-structural details, such as dentin walls, core root filling material and sealer, tiny gaps, voids or cracks, making them particularly ideal for precise assessment of the filling, sealing conditions, and the properties of the filling materials [21,22,23,24,25]. Nevertheless, high resolution imaging is primarily used as tool for RCT research rather than routine clinical practice, due to their ex vivo (Both imaging modalities require tooth extraction during scanning, and microscopy requires sectioning the tooth sample), time-consuming, costly, and high-radiation (for micro-CT) nature. Ultra-high field magnetic resonance imaging (MRI) has also been applied in root canal imaging, particularly for dental material research [26].

Figure 5 shows a volume rendering of a micro-CT scan of a treated premolar, where various canal structures and materials are clearly distinguished. Beyond visualization, micro-CT provides ground-truth three-dimensional morphological information that supports more accurate treatment planning and diagnostic research, enables population-level comparison of anatomical variability, and offers a benchmark for validating clinical imaging and computational methods. The 3D morphologies of root canal systems and its sub-structures can be segmented from imaging data and analyzed quantitatively. Quantitative morphological analysis at ultra-high-resolution in 3D is particularly significant in the context of this review, as computational methods, such as root canal segmentation [6,27,28] and void detection [29,30,31], are developed and applied using micro-CT data. Note that some studies use CBCT to assess voids generated by air bubbles or irrigant entrapment beyond filling materials [32]. However, a key challenge remains: how can findings derived from micro-CT be effectively translated into routine dental imaging techniques, such as X-ray and CBCT, for clinical practice? One example involves investigating the relationship between treatment failure factors, such as voids and gaps, and the 3D morphology of the root canal, then correlating these findings with X-ray and CBCT images [33]. Another example focuses on determining how many voids and gaps can result in treatment failure and exploring their correlation with clinical outcomes to identify thresholds for successful treatment, e.g., a study by Liang, Y.H. et al. [34] found that rhe presence of radiographically detectable voids in root canal fillings was linked to poorer treatment outcomes. A third example examines the effect of different filling materials and techniques under microscopy, with the goal of applying these insights to improve clinical practices [35,36]. We anticipate that computational methods bridging such a gap, such as those presented by Lamira, A. et al. [4], Wu, W. et al. [5] and Lin, X. et al. [6], will be crucial for enabling more informed RCT planning, and more objective quality assessments and prognosis predictions, compared to the status quo. Effective translation from research to practice is particularly important in the context of RCT.

## 3. Computational Approaches in Root Canal Treatment: A Review of Methods

As shown in our taxonomy (Figure 2), computational approaches can be applied throughout the RCT life cycle, including planning, treatment quality evaluation and assurance, prognosis prediction, follow-ups, as well as documentation (potentially using LLMs [37]). The planning and root canal preparation phases involve analyzing canal shape, orientation, size, curvature, and anatomical variations (e.g., lateral canals, branches), which is crucial for achieving optimal cleaning, shaping, sealing, and filling while reducing procedural risks like instrument fracture and canal perforation [38,39]. After treatment, the filling quality, which is essential for the long-term success of the treatment, needs to be assessed. Quantitative measurements like the distance between root filling and root apex, filling homogeneity or density (influenced by the presence of voids or gaps), and taper consistency [40,41,42], are suggested as indicators of treatment quality. Note that volume change in pulp chamber is also a quality indicator for Regenerative Endodontic Procedures (Regendo) [43]. Segmenting the root canal system in 3D image data such as micro-CT, including the pulp, sealer, core filling material, and other relevant sub-structures, is a prerequisite for these quantitative analyses as well as the development of computational methods for automatic treatment quality assessment.

**Figure 6:**
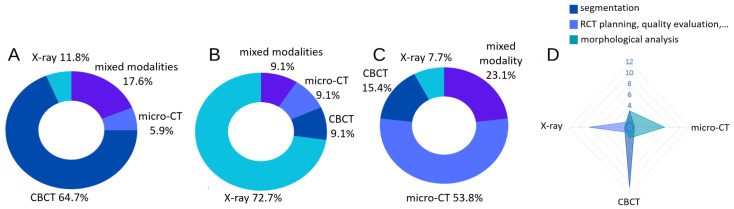
The number of papers using a specific dental imaging modality across the three taxonomy categories: segmentation (**A**), RCT planning, quality evaluation and prognosis prediction (**B**), and morphological analysis (**C**). A radar plot of imaging modality and taxonomy categories is shown in (**D**).

Numerous computational methods, including automatic segmentation, classification, and landmark localization, have been developed with a focus on teeth and crowns [44,45,46,47,48,49], with some extending to the tooth root [50,51,52,53]. However, significantly fewer methods have been applied to root canals, especially those that have been treated with gutta-percha filling. In this section, we review computational methods related to the root canal system and categorize them according to the stages they are applied in RCT as well as their technical relevance: (1) Segmentation; (2) Treatment planning, quality evaluation, and prognosis; (3) Morphological analysis. Figure 6 shows the number of papers using a specific dental imaging modality across the three categories. The overlap between Section 3.1 (segmentation), Section 3.2 (clinical management) and Section 3.3 (morphological analysis) is significant and can be understood intuitively: the root canal system’s morphology is obtained through segmentation, and analyzing this morphology along with quantitative measurements at different stages provides insights for RCT clinical management. These methods are summarized in Table 3, Table 4 and Table 5. Wang, Y.C.C. et al. [54] developed a pipeline that combines CNN-based object detection and semantic segmentation to identify and analyze root canal fillings, as well as other dental conditions like caries and implants. Ourang, S.A. et al. [55] provides an overview of how artificial intelligence (AI) can help with endodontic tasks. Refer to Table 6 for an overview of the technical terms used in the following discussion.

### 3.1. Segmentation

**Figure 7:**
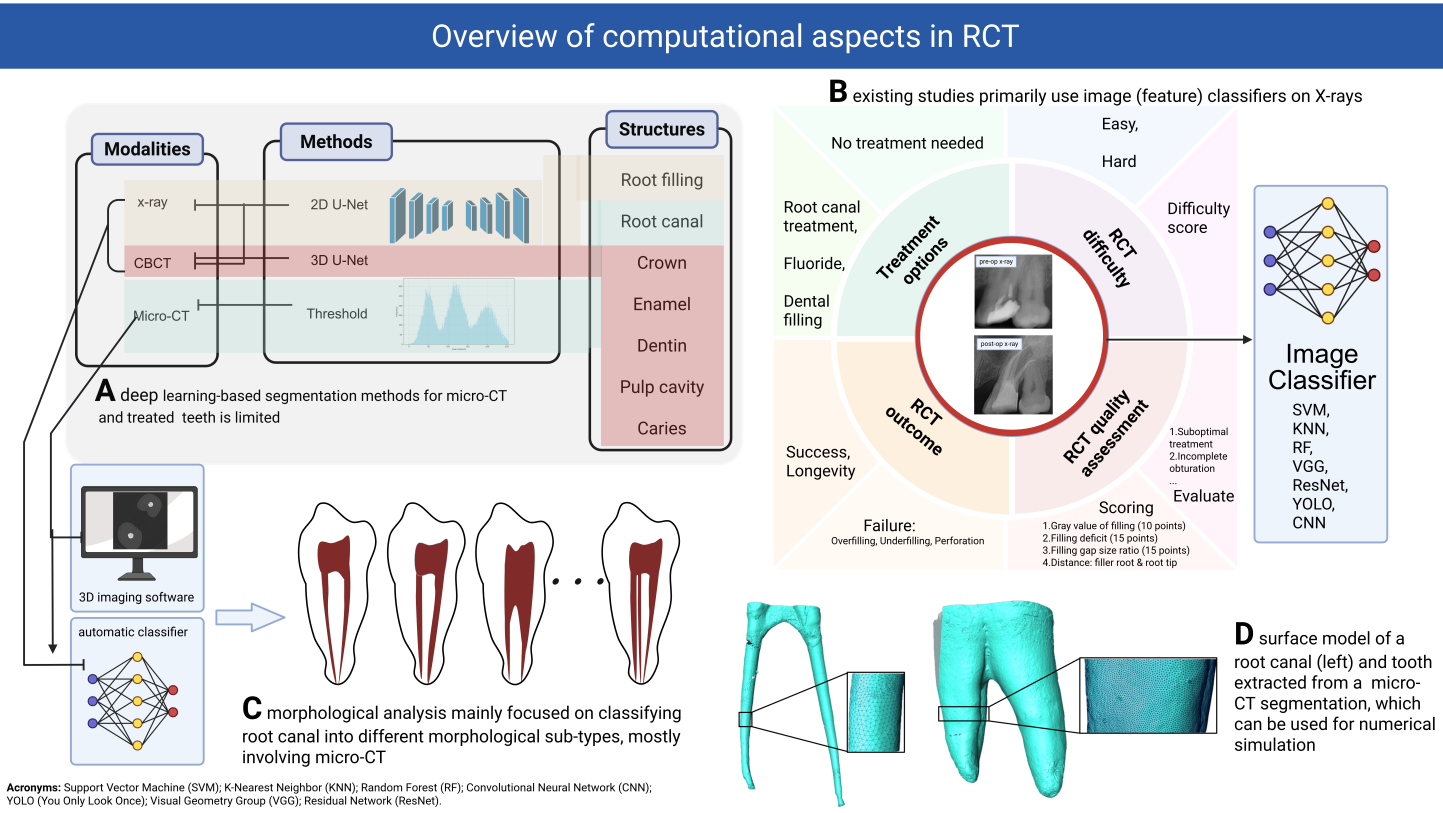
Overview of the computational aspects in RCT, including (**A**) segmentation of different tooth structures; (**B**) prediction of treatment options, RCT difficulty, treatment quality, treatment outcome and prognosis; (**C**) analysis of root canal morphological variations, and (**D**) numerical simulation based on the surface model of tooth and root canal. This figure is created using BioRender.

This section reviews segmentation methods that focus on the root canal and its sub-structures, including the pulp, as well as sealer and core filling material (where applicable, in the case of treated teeth). An overview of these methods is provided in Table 3 and Figure 7A. Most studies segment the root canal in conjunction with other dental sub-structures, such as the crown, dentin, or the full tooth, while only a few specifically focus on the root canal. Two common technical challenges in root canal segmentation identified by these methods are (i) the ROI occupies only a small portion of the large CBCT image [92], and (ii) the difficulties posed by high-resolution imaging [61], which complicates computational processing. To address these challenges, techniques such as ROI cropping and coarse-to-fine segmentation are frequently employed in these methods.

#### 3.1.1. Joint Segmentation of Tooth and Its Sub-Structures

Dumont, M. et al. [57] and Deleat-Besson, R. et al. [56] aimed to create a holistic tooth segmentation including both the crown and root canal for analyzing the full tooth anatomy, by using two imaging modalities, i.e., CBCT for root canals and intra-oral scans for dental crowns. To this end, a 2D U-Net was trained to segment the root canal area from CBCT slices, and another modified U-Net was adopted to segment dental crowns from intra-oral scans. The authors further proposed an algorithm for merging the segmented root canals with crowns. Wang, Y. et al. [58] introduced 3D PulpNet and DentalNet, designed for the segmentation of the pulp chamber, root canals, and crown area in CBCT images. Specifically, DentalNet was used to identify the tooth region, which then defined a ROI to refine the input for subsequent 3D PulpNet segmentation. The authors also assessed their methods through two clinical case studies that required 3D models of the tooth and root canal. Duan, W. et al. [59] introduced a two-phase method for tooth and pulp segmentation. In the first phase, a region proposal network (RPN) is used to identify the ROI containing the tooth and root canal from CBCT images. In the second phase, a 3D U-Net is used to segment the two areas sequentially, with the segmented tooth region defining a ROI for pulp segmentation, similar to the approach introduced by Wang, Y. et al. [58]. The study also differentiated between single-rooted and multi-rooted teeth and introduced a smoothness regularization in the loss function to address the challenge of defining pulp boundaries. Unlike other studies that involve two separate steps for the tooth and root canals, Li, S. et al. [61] proposed a method to segment the two regions simultaneously. A coarse-to-fine framework was proposed to handle high-resolution CBCT data. Initially, the tooth and root canal region was segmented jointly on down-sampled (coarse) images, which defines a ROI for the original high-resolution CBCT data. Then, the cropped ROI was fed into a second network based on transformer to obtain the high-resolution segmentation masks of both regions. Zhang, X. et al. [62] used a conditional generative adversarial network (cGAN) to segment multiple tooth sub-structures from CBCT images with acute pulpitis, including the enamel, dentin, pulp, crown, caries and root canals. Lin, X. et al. [6] segmented the tooth, pulp cavity, pulp chamber and root canals jointly using a 2D U-Net. The authors collected both CBCT and micro-CT scans of the same teeth. Ground truth segmentation were obtained in two ways: manual segmentation from CBCT images by an expert and threshold-based segmentation from micro-CT. The two sets of segmentations were then registered and used to train the U-Net separately on CBCT images as labels. Results showed improved accuracy using the micro-CT ground truth. Michetti, J. et al. [28] evaluated an adaptive threshold method for tooth and root canal segmentation based on CBCT images, and compared the results with high-quality segmentations from micro-CT after a registration step, in terms of volume, area and Feret’s diameter. CBCT segmentations showed slight under-estimation. Ari, T. et al. [66] and Gardiyanoğlu, E. et al. [67] targeted treated teeth with fillings and used a 2D U-Net to segment various dental sub-structures from radiographs. Tan, M. et al. [64] presented a three-stage deep learning framework for automatically segmenting 3D teeth and their sub-structures (enamel, pulp, dentin) from CBCT images, utilizing centroid detection, a tooth segmentation network, and an attention-based hybrid feature fusion mechanism.

#### 3.1.2. Root Canal Segmentation

Zhang, J. et al. [60] employed a multi-task 3D U-Net to segment both the root canal region and the contour of the root canal from CBCT images. Machado, J. F. [65] acquired image data of a set of extracted teeth using both CBCT and micro-CT, and compared the root canal volume obtained from each imaging modality following a threshold-based root canal segmentation using the Fiji software. The study revealed a low agreement between the volume measurements obtained from CBCT and micro-CT. Haberthür, D. et al. [27] used an automated Otsu thresholding method for root canal segmentation from micro-CT. The extracted root canals were further refined by removing speckles and filling small holes. Slim, M.L. et al. [68] and Santos-Junior, A.O. et al. [69] employed a two-stage approach, where each stage utilized a 3D U-Net for segmenting the pulp cavity from molar and premolar teeth in CBCT images. The initial U-Net generated preliminary segmentations, which were subsequently refined by the second U-Net at full resolution. The segmentation of root canals from CBCT or micro-CT images is commonly converted into 3D models for finite element analysis (FEA)- or boundary element method (BEM)-based simulations (e.g., Figure 7D), which are used to assess stress distribution [93,94,95,96], durability [97], fatigue [98], fracture risk [99] or evaluate the root canal preparation quality [100]. Additionally, 3D root canal structures segmented from micro-CT or CBCT scans can be utilized for 3D printing or as digital models in various dental applications. These include guided endodontic treatment [101,102,103], autotransplantation [104], and dental education, such as anatomy teaching with 3D-printed teeth [105,106] or immersive learning through virtual and mixed reality [107,108].

### 3.2. Treatment Planning, Quality Evaluation and Prognosis

**Figure 8:**
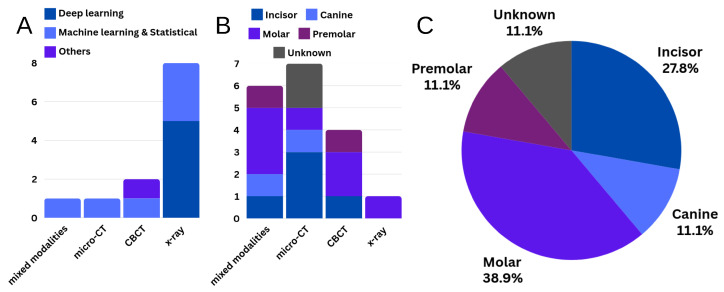
(**A**) Frequency of different types of methods (Deep Learning, Machine Learning and Statistical, others) used for treatment planning, quality assessment, and outcome prediction. (**B**) Frequency of different tooth types (e.g., incisor, canine, molar, premolar) analyzed across imaging modalities for morphological analysis. (**C**) Percentages of tooth types used in morphological analysis.

Computational methods for RCT planning, quality evaluation, and prognosis prediction are summarized in Table 4 and Figure 7B. These methods typically frame the tasks as classification or regression problems, where traditional machine classifiers, such as support vector machine (SVM), k-Nearest Neighbors (KNN), gradient boosting machines (GBM) and random forest (RF), or convolutional neural networks (CNN)-based methods, such as VGG and ResNet, are employed to categorize dental images or measurements into predefined treatment options or outcome groups. In traditional machine learning approaches, morphological features are typically extracted manually from the root canal region on unsegmented images, though some studies first perform root canal segmentation before feature extraction [41,42,43,58]. In contrast, deep learning-based methods implicitly learn these morphological features directly from dental images. Figure 8A shows the frequency of different methods used in these studies. Refer to Figure 7B for a brief overview of CNN-based methods in RCT clinical applications.

One study, i.e., Pinto, J. C. [70], assessed the effect of voxel size (5 μm, 10 μm, 20 μm) of micro-CT images on the evaluation of root canal preparation in terms of the percentage of root canal volume increase, debris and the root canal surface of uninstrumented area. Two instrumentation system, ProDesign Logic and HyFlex EDM, were adopted to prepare the mesial root canals of mandibular molars. Student’s *t*-test and ANOVA tests showed that no statistical differences were found in the measurements between the two systems. Micro-CT imaging at 5 μm voxel size demonstrated higher accuracy for assessing uninstrumented root canal surfaces, while voxel size did not significantly impact the evaluation of other variables.

#### 3.2.1. Treatment Planning and Recommendation

Bouchahma, M. et al. [72] used a CNN-based image classification network to predict treatment options, i.e., fluoride, filling and RCT from X-rays for dental decays (caries). Similarly, Latke, V. et al. [73] addressed the problem of classifying X-rays into different treatment options, using SVM and KNN. Choudhari, P. et al. [74] provided an overview of methods developed for detecting dental diseases such as dental decay and root infection, and predicting the corresponding endodontic treatments. Karkehabadi, H. et al. [79] adopted CNN-based classifier to predict the difficulties of RCT (easy, hard) and a difficulty score based on X-rays. The difficulty level of each case is determined by dentists and endodontists according to the American Association of Endodontists (AAE) guidelines.

#### 3.2.2. RCT Quality Evaluation, Outcome Prediction, Prognosis and Follow-Ups

Lamira, A. et al. [4] analyzed the anatomy of mesial canals with isthmuses in mandibular molars before and after RCT using both CBCT and micro-CT images. Their evaluation focused on four aspects: debris presence, root perforation, filling quality, and 2D parameters (area, diameter, perimeter, roundness). Statistical analysis, including the Tukey test, revealed that while CBCT showed strong agreement with micro-CT in measuring 2D parameters of prepared and treated root canals, its accuracy in detecting debris, root perforations, and partially filled isthmuses was limited. Zhou, Y. et al. [71] used ResNet to predict a quantitative score that reflects the treatment quality based on X-ray images. Hasan, H.A. et al. [75] used the YOLO network [109] to classify the post-operative X-rays into different outcome groups, such as suboptimal treatment, incomplete or complete obturation. Choudhari, P. et al. [76] utilized machine learning classifiers, such as SVM and Bayes, to predict RCT failure types, including over-filling, under-filling, and perforation, as well as the treatment’s longevity, defined as the duration for which it remains effective. Similarly, Qu, Y. et al. [78] identified eight key features from CBCT images, including patient age and sex, tooth type (maxillary, mandibular, anterior, molar, premolar), number of root canals, lesion size, bone defect type, and the density and length of root filling. Using these features, GBM and RF were employed to classify treatment outcomes, which were assessed one year post-treatment following the criteria established by Rud, J. et al. [110]. Bennasar, C. et al. [77] conducted a study on prognosis prediction that relied solely on preoperative variables, including patient demographics, medical history, clinical symptoms, and various X-ray measurements and findings. Using these inputs, machine learning classifiers were employed to categorize cases as either success or failure, with outcomes determined based on each patient’s nine-year follow-up data. Liu, J. et al. [42] and Peng, G. et al. [41] proposed a quantitative approach for evaluating RCT quality, incorporating root canal filling segmentation using U-Net and a scoring system based on criteria such as the compactness of the filling material and distance measurements (e.g., the gap between the root filling and root apex). ResNet was then employed to predict multiple scores, each reflecting the extent to which a specific criterion was met. These scores demonstrated a strong agreement with manual endodontic assessments. Notably, unlike other studies, Liu, J. et al. [42] and Peng, G. et al. [41] explicitly defined the ROI as the root canal and filling area through segmentation, rather than analyzing entire dental images (X-ray or CBCT). Additionally, Shetty, H. et al. [43] assessed pulp volume changes, an indicator of tissue removal before and after RCT, using semi-automatic segmentation tools such as 3D Slicer.

### 3.3. Morphological Analysis

Studies investigating the morphology of the root canal system are summarized in Table 5 and Figure 7C. Most research in this area has focused on classifying root canal configurations (RCC), which describe anatomical variations in the shape, number, and branching patterns of root canals. Different RCCs can impact the complexity of RCT, with features like lateral canals and isthmuses posing challenges for thorough cleaning and sealing. Other studies have focused on either morphological measurements, primarily using (computer-aided) manual analysis, or image super-resolution techniques. Figure 8B,C shows the frequency of different tooth types used for morphological analysis.

#### 3.3.1. Root Canal Morphology Classification and Measurements

RCC is an important consideration in both endodontic procedures and the development of computational methods. Over time, various classification systems have been introduced, including Weine’s [111,112], Vertucci’s [113,114], and Briseño’s [115], along with more recent approaches that incorporate additional morphological variations identified through advanced dental imaging [80]. Figure 3G illustrates different morphological variations in root canals. Haberthür, D. et al. [27] segmented the root canals from micro-CT images employing Otsu thresholding, and subsequently classified RCC based on four axial slices at predefined locations according to the Briseño classification [115]. Wolf, T.G. et al. [84] analyzed the morphologies of mandibular incisor root canals of a German population and reported statistics in Briseño’s RCC, existence of foramina and frequency of accessory canals. The group later reported the statistics of canine and incisor root canals on a Swiss-German population [82,83]. Wu, W. et al. [5] used CNN-based image classifiers like VGG and ResNet, to implicitly learn morphological features of the second molar root canals from X-rays and classify them into three morphological types, including merging, symmetrical and asymmetrical. The ground truth morphological type was determined from the corresponding paired micro-CT scans. Hiraiwa, T. et al. [86] used AlexNet and GoogleNet to identify X-rays with an extra root canal for first molar teeth. The existence of an extra root is determined from the corresponding paired CBCT images. Lyu, L. et al. [81] studied the root canal morphology of the central and lateral maxillary incisors obtained from micro-CT images, and measured the canal volume, surface area, circumference, root canal length, roundness mesial-distal diameter, etc. The measurements for the central and lateral incisors were qualitatively and quantitatively compared and discussed.

#### 3.3.2. Super-Resolution

As discussed in Section 2.2, a resolution gap exists between clinical routine imaging (e.g., X-ray, CBCT) and high-resolution research imaging (e.g., micro-CT). Developing computational methods to enhance the resolution of routine imaging is highly desirable. In Hatvani, J. et al. [87], CBCT and micro-CT scans of ex vivo tooth samples were acquired and registered using 3D Slicer. The paired CBCT and micro-CT images were then used to train a 2D U-Net model for slice-wise super-resolution. The effectiveness of resolution enhancement was assessed by segmenting root canals on the enhanced CBCT images and comparing the results to those obtained from micro-CT. Likewise, Ji, Y. et al. [90] used Basicvsr++ [116] to learn the resolution mapping from CBCT to micro-CT images. Their study also demonstrated improved identification of MB2 canals using the enhanced CBCT data. Additionally, volume measurements obtained from root canal segmentations on the enhanced CBCT images were compared to those from the gold standard, i.e., micro-CT, to test the agreement between the two imaging modalities. Sfeir, R. et al. [88] developed a linear model to enhance CBCT image resolution, based on the assumption that low-resolution CBCT data represents a degraded and downsampled version of its high-resolution counterpart. The degradation was modeled using a decimation matrix, a deconvolution operator, and additive Gaussian noise. High-resolution images were reconstructed by solving a regularized inverse problem with optimization and total variation regularization, addressing the ill-posed nature of the super-resolution task. The method’s effectiveness was evaluated through root canal segmentation by comparing volume measurements from the enhanced CBCT images with those from micro-CT. This approach was later extended to 3D [89].

## 4. Discussion and Conclusions

In this review, we presented an in-depth discussion of selected computational methods relevant to RCT, organized according to a comprehensive taxonomy that spans every stage of the RCT life cycle. In this section, we summarize the current state of these methods, highlight the existing methodological gaps and challenges, and offer suggestions for future research, along with practical recommendations for the implementation of computational approaches based on our experience in this field.

### 4.1. Current State

#### 4.1.1. Segmentation

**Figure 9:**
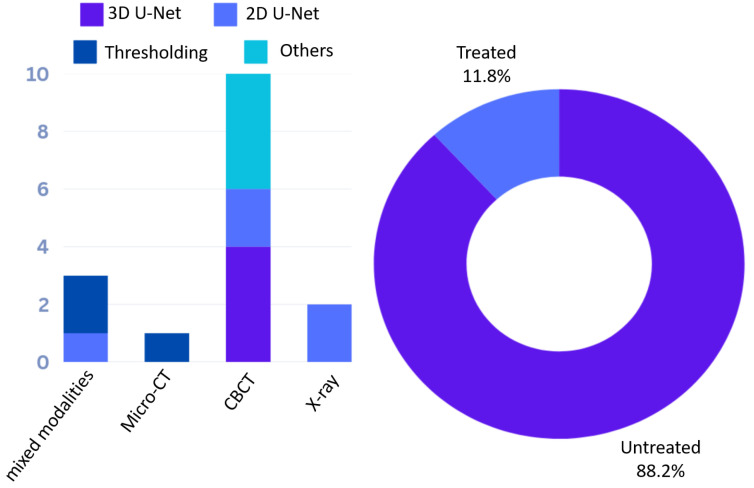
The bar plot on the left illustrates the number of different segmentation methods (3D U-Net, 2D U-Net, Thresholding, and others) used for each imaging modality (micro-CT, CBCT, X-ray, mixed). The Donut plot on the right displays the percentage of treated versus untreated teeth included in these studies.

While X-ray is the most commonly used imaging modality in routine practice for RCT, most segmentation methods are developed using CBCT images, driven by the need for 3D analysis. Most methods are based on 2D or 3D U-Net architectures, targeting multiple tooth regions, besides the canals. Additionally, according to Figure 9, the majority of studies use untreated teeth, with fewer addressing treated teeth with root canal filling, typically using radiographs [66,67]. Furthermore, Table 3 shows that only a few studies have used micro-CT for root canal segmentation, with threshold-based segmentation methods applied. Novel methods that leverage accurate ground truth segmentations from micro-CT to supervise training on CBCT have been proposed [6].

#### 4.1.2. Treatment Planing, Quality Evaluation and Prognosis

Treatment planning, quality evaluation, and prognosis prediction are typically framed as classification or regression problems. Most methods in this category focus on treatment quality evaluation and prognosis prediction based on X-rays of treated teeth, with fewer studies employing CBCT or micro-CT images—a trend that aligns with our discussion in Section 2 on the use of different imaging modalities in clinical practice. A key highlight is the methods that investigate a correlation between pre-operative root canal morphology and prognosis outcomes [77].

#### 4.1.3. Morphological Analysis

Micro-CT is the most commonly used imaging modality for 3D quantitative morphological analysis, which aligns with our discussion in Section 2. Existing methods focus on classifying RCC and measuring morphological features, both of which are important factors to consider during the planning, treatment and evaluation processes. Classic morphological analysis techniques such as Statistical Shape Models (SSMs), although not yet widely applied to micro-CT datasets in endodontic research, offer valuable tools for quantifying population-level anatomical variation in root canal systems. A typical SSM workflow involves establishing spatial correspondence across a cohort of canals, followed by dimensionality reduction techniques such as Principal Component Analysis (PCA) to identify dominant modes of variations, such as canal curvature. These statistical descriptors provide a quantitative basis for characterizing anatomical complexity, which is closely associated with treatment difficulty and the likelihood of procedural failures.

#### 4.1.4. Critical Evaluation

The computational methods for dental image segmentation, classification, and morphological analysis discussed in Section 3 generally demonstrate strong performance at the research level. However, evidence of their clinical applicability remains limited. A major challenge is their limited generalizability to real-world data, as most models are trained and evaluated on controlled datasets that are retrospective, small in sample size, and derived from a single institution. For instance, a U-Net trained for micro-CT segmentation may perform poorly when applied to micro-CT data acquired with different scanners, resolutions, or imaging parameters. In addition, commonly used evaluation metrics, such as the Dice Similarity Coefficient (DSC), may not reflect clinical priorities. For example, segmentation errors in the apical region are far more consequential for prognosis than errors in the middle third, yet most metrics assign them equal weight. These issues of domain generalization and metric misalignment are well-recognized barriers to clinical translation in medical image analysis [117]. Moreover, the limited interpretability of deep learning models remains a significant obstacle to gaining clinician trust and supporting decision-making. Based on these considerations, future focus should also be placed on establishing multi-center standardized datasets with privacy-preserving collaboration frameworks such as federated learning, integrating AI tools into existing CBCT and clinical software systems, and developing human-in-the-loop workflows to fully bring these computational algorithms into clinical practice.

### 4.2. Future Direction, Challenges and Practical Recommendation

Future research in computational methods for RCT should address several under-explored areas, including (i) defects detection and defect type classification; (ii) segmentation of treated root canals with core filling and sealing materials based on micro-CT; (iii) Development of explicit, data-driven approaches for 3D morphological analysis of the root canal system, including the core filling material and sealer of treated teeth and any defects, using high-resolution imaging techniques such as micro-CT, to examine the relationship between the morphology of the pre- or post-operative root canal system and treatment outcomes; (iv) Application of insights gained from high-resolution imaging to routine clinical practice.

#### 4.2.1. Defect Detection and Classification

As shown in Table 1, defects can occur at different stages of RCT and can arise from various causes, including instrumentation (cracks, root perforation), insufficient cleaning, filling and sealing due to complex canal morphology (gaps, voids, debris), and material-related factors (pores). Computational methods that automatically locate these defects in treated root canals can enhance the accuracy and efficiency of treatment assessment, yet such methods remain largely absent in existing research. Classifying defect types to distinguish their causes can help improve the corresponding procedures. It is recommended that micro-CT be used in the development process to prevent the oversight of small defects that may not be well depicted on X-ray or CBCT images.

#### 4.2.2. Micro-CT Based Segmentation

Given that state-of-the-art segmentation algorithms, such as nn-UNet [118], utilize large annotated datasets and are based on supervised learning, future research should focus on developing learning-based segmentation methods specifically targeting treated teeth with sealer and filling material using micro-CT (Figure 9). However, challenges such as acquiring large volumes of micro-CT data, performing annotations, and addressing the computational difficulties posed by ultra-high-resolution imaging data (e.g., a single micro-CT scan may occupy more than 50 GB with a 2400 × 2500 × 3800 resolution) must be overcome. Two-dimensional slice-wise training and partial annotations, where only specific slices of the micro-CT scans are annotated, could help alleviate issues related to annotation efficiency and memory requirements.

#### 4.2.3. Explicit, Learning-Based Morphological Analysis

Current approaches in the category of morphological analysis primarily rely on deep learning models to implicitly learn morphological features of the root canal system from grayscale dental images. However, explicit morphological analysis of the root canal system, particularly those using data-driven techniques, remains a significant research gap. Methods like statistical shape modeling, which have been successfully applied to various human anatomical structures, such as the skull [119], knee [120,121], spine [122,123], aorta [124,125], heart [126,127], and brain [128], have not yet been explored for the root canal system in endodontic research. This is further complicated by the lack of exploration into more recent learning-based methods such as neural radiance fields (NeRF) [129], diffusion models [130], and Gaussian splatting [131]. A key challenge is the acquisition of high-resolution morphological data of the root canal system, which requires costly micro-CT imaging. Sparse methods, such as those discussed by Li, J. et al. [92], could potentially help mitigate computational limitations when working with high-resolution data for morphological analysis.

#### 4.2.4. Translation of Research Insights into Clinical Practice

While micro-CT is ideal for 3D morphological analysis, it is not practical for routine clinical use (as discussed in Section 2). There is a strong demand for computational methods that can transfer the insights gained from 3D morphological analysis of the root canal system using micro-CT to routine dental imaging, such as X-rays and CBCT. Our review highlights that one common approach to achieving this is by pairing routine imaging data with micro-CT data and learning a mapping between the routine images and a ground truth derived from micro-CT. This ground truth can be either a morphological feature [5], a segmentation [6] or micro-CT itself (for super-resolution) that cannot be accurately determined from routine imaging. The learned mapping can be applied to routine images to carry out tasks such as segmentation and morphological analysis with micro-CT-level precision.

#### 4.2.5. Overview of Computational Tools and Software

To support readers without a technical background, we provide in this section a non-technical overview of the computational methods relevant to RCT (Section 2). These methods can be broadly grouped into three categories: general image processing, AI-enabled automatic image segmentation, and AI-based classification. For example, automatic segmentation tools take dental images such as micro-CT, CBCT, or X-ray scans as input and generate segmented structures (e.g., root canals, pulp, or gutta-percha fillings), which can then be quantitatively analyzed for defects, volumes, or spatial distribution. Common AI-based segmentation approaches include U-Net [132] and its widely adopted variant nnU-Net [118]. Image-processing software with graphical interfaces, such as 3D Slicer (https://www.slicer.org/) and ITK-SNAP (https://www.itksnap.org/), provides assisted segmentation, visualization, and analysis capabilities, and some platforms also integrate automatic segmentation modules. For example, the nnU-Net medical image segmentation framework has been integrated into 3D Slicer as an extension, allowing users to run trained models and visualize segmentation results directly within the Slicer interface, thereby enabling a seamless workflow for image segmentation and analysis (Refer to https://github.com/gaudot/SlicerDentalSegmentator/, accessed on 17 November 2025 and https://github.com/KitwareMedical/SlicerNNUnet, accessed on 17 November 2025). Deep learning classifiers, typically built on CNN architectures, can analyze dental images such as X-rays to predict treatment outcomes or recommend treatment options by categorizing image features automatically. Commonly used models include ResNet, VGG, AlexNet, and GoogleNet, which form the backbone of AI-assisted diagnostic systems in RCT. Many commercial and research-focused dental software solutions integrate these functions into user-friendly interfaces to support clinicians without technical expertise.

#### 4.2.6. Manuscript Preparation

During manuscript preparation, generative AI (ChatGPT, version GPT-5.1, OpenAI) were used only for linguistic refinement. Specifically, the authors provided manually written text and asked the tool to suggest improvements to grammar, wording, and sentence structure. ChatGPT’s suggestions were then carefully reviewed, verified, and manually corrected where needed by the lead authors throughout both the initial drafting and revision stages. Finally, all authors independently reviewed the full manuscript manually and suggested additional edits where necessary.

## Figures and Tables

**Table 1:** A list of dental terms related to Root Canal Treatment (RCT). For additional clarification and illustrations, see Figure 3.

Terminology	Explanation
Root canal	The pulpal space within the root(s)
Canal orifice	The opening of a root canal
Apical foramen	The root tip opening where nerves, vessels enter the tooth
Root canal configuration	The shape, number, and branching of root canals
Single- or multi-rooted teeth	Teeth with a single or multiple roots (Molars typically have multiple roots, while incisors and canines have a single root. Note that a root can contain multiple canals, depending on the tooth type and canal branching.)
Coronal, middle apical	Parts of the tooth root from crown to root tip
Lateral (accessory) root canal	Canals branching from the main root canal
Isthmus	An irregular connection between two canals in a root
MB2 canal	The second canal within the MB root of maxillary/upper molars
Pulp stone	Calcified deposit found within the dental pulp
Defects	A broad term referring to any imperfections in root canal filling
Voids and Pores	Entrapped air inside the filling materials
Debris	Leftover tissue and bacteria after pulp removal
Gaps & Delamination	Separation of layers between sealer, dentin, gutta-percha

**Table 2:** Comparison of Imaging Modalities in RCT.

Modality	Resolution	Dimension	Feature	Radiation	Usage	Micro-Structure
X-ray	high	2D	in vivo and non-destructive	low	clinical routine	Limited
CBCT	moderate	3D	in vivo and non-destructive	moderate	clinical routine	Large voids or gaps, root apex
Microscopic	ultra-high	2D	ex vivo and destructive	none	research	Surface morphology and material-dentin interaction
Micro-CT	high	3D	ex vivo and non-destructive (While being ex vivo, micro-CT is considered ’non-destructive’, with respect to the imaged sample, i.e., tooth.)	very high	research	3D evaluation of material distribution, voids, and gaps
PCE micro-CT	high	3D	ex vivo and non-destructive	very high	research	Finer micro-structure details with varying contrasts
MRI	moderate	3D	ex vivo and non-destructive	non-ionizing	research	Clear distinction of dental materials, e.g., dentin, sealer and gutta-percha

**Table 3:** Summary of Segmentation Methods in RCT.

Paper	Modality	Method	Target	Notes
Deleat-Besson et al. [56] (2020)	CBCT	2D U-Net	Crowns and Root canals	Match crowns with the respective root canals
Dumont et al. [57] (2020)	CBCT	2D U-Net	Crowns and root canals	Match crowns with the respective root canals
Wang et al. [58] (2023)	CBCT	3D PulpNet	Tooth and root canals	Jointly segment teeth and root canals
Duan et al. [59] (2021)	CBCT	3D U-Net	Tooth and pulp cavity	Single and multi-rooted teeth
Zhang et al. [60] (2021)	CBCT	3D U-Net	Root canals	Root canal area and contour
Li et al. [61] (2023)	CBCT	transformer	Tooth and root canals	Jointly segment teeth and root canals
Zhang et al. [62] (2022)	CBCT	cGAN	Caries, enamel, dentin, dental pulp, crown, root canal	Segment multiple tooth sub-structures
Harris et al. [63] (2023)	CBCT	dental anatomy- based heuristics	Tooth and pulp	Single and multi-rooted teeth
Tan et al. [64] (2024)	CBCT	Attention-based deep learning	Enamel, pulp and dentin	Robust against dental artifacts like metal and calcification
Lin et al. [6] (2021)	CBCT + Micro-CT	2D U-Net	Tooth and pulp cavity	Train U-Net using manual labels from CBCT and threshold-based labels from micro-CT
Michetti et al. [28] (2017)	CBCT + Micro-CT	Thresholding	Root canals	Comparison of CBCT and Micro-CT segementation
Machado et al. [65] (2019)	CBCT + Micro-CT	Thresholding	Root canals	Comparison of CBCT and Micro-CT segmentation
Haberthür et al. [27] (2021)	Micro-CT	Otsu threshold and island removal	Root canals	Segment the root canal and analyze the morphology
Ari et al. [66], Gardiyanoğlu et al. [67] (2022, 2023)	X-ray	2D U-Net	Various dental structures (Caries, implants, lesion, crown, pulp, root canal filling)	Jointly segment various structures of treated teeth
Slim et al. [68], Santos-Junior et al. [69] (2024, 2025)	CBCT	3D U-Net	Pulp cavity	Pulp cavity of molar and premolar teeth

**Table 4:** Summary of Computational Methods Focusing on Treatment Planning, Treatment Quality Evaluation and Prognosis in RCT.

Paper	Modality	Method	Notes
Pinto et al. [70] (2023)	Micro-CT	Statistical analysis (Student’s *t*-test and ANOVA tests)	Effect of micro-CT voxel size on the evaluation of root canal preparation
Lamira et al. [4] (2022)	Micro-CT and CBCT	Statistical analysis (kappa coefficient, variance, Tukey test)	Comparison of CBCT- and micro-CT-based RCT quality evaluation
Zhou and Zhang [71] (2021)	X-ray	ResNet	Generate a quantitative score based on treated images to reflect treatment quality
Bouchahma et al. [72] (2019)	X-ray	CNN-based image classification	Predict treatment options for dental decay
Latke and Narawade [73] (2023)	X-ray	SVM, KNN	Predict treatment options for dental decay
Choudhari [74] (2022)	-	-	Detect dental diseases and recommend treatment
Hasan et al. [75] (2023)	X-ray	YOLO network	Predict RCT outcome
Choudhari et al. [76] (2024)	X-ray	logistic regression, Bayes, SVM	Predict RCT failure types and longevity
Bennasar et al. [77] (2023)	X-ray	RF, KNN	Predict prognosis—success or failure, using pre-operative features
Qu et al. [78] (2022)	CBCT	GBM, RF	Predict prognosis—outcome one year after treatment
Karkehabadi et al. [79] (2024)	X-ray	VGG, ResNet and Inception	Assess RCT difficulty
Liu et al. [42], Peng et al. [41] (2022, 2024)	X-ray	U-Net, ResNet	Quantitative evaluation of RCT quality based on segmented canal and filling area
Shetty et al. [43] (2021)	CBCT	OsiriX MD and 3D Slicer and Materialize MiniMagics	Pulp volume estimation before and after RCT

**Table 5:** Summary of morphological analysis methods in RCT.

Paper	Modality	Method	Target	Notes
Haberthür et al. [27] (2021)	Micro-CT	Briseño classification	Root canals	RCC Classification using four slices
Ahmed et al. [80] (2017)	Micro-CT	-	Root canals	A new RCC scheme
Lyu et al. [81] (2024)	Micro-CT	Morphological measurement	Incisor root canals	Root canal measurement, e.g., length, volume, surface area
Wolf et al. [82] (2021)	Micro-CT	3D imaging software	Canine root canal	Root canal classification and measurement of the extracted teeth of a Swiss-German population
Wolf et al. [83] (2024)	Micro-CT	3D imaging software	Incisor root canal	Root canal classification and measurement of the extracted teeth of a Swiss-German population
Wolf et al. [84] (2020)	Micro-CT	3D imaging software	Incisor root canals	Root canal classification and measurement of the extracted teeth of a German population
Wu et al. [5] (2024)	Micro-CT and X-ray	VGG, ResNet, EfficientNet	Second, molar root canals	Classification of second molar morphology types based on 2D X-rays, using 3D micro-CT as ground truth
Karobari et al. [85] (2022)	Micro-CT	-	Anterior and third molar toot canals	A systematic review of root canal morphology classification
Hiraiwa et al. [86] (2019)	CBCT and X-ray	AlexNet and GoogleNet	First, molar distal root canals	Classification of root canal morphology based on radiographs using CBCT as ground truth
Hatvani et al. [87] (2018)	Micro-CT and CBCT	2D U-Net	Incisor, canine, premolar, and molar root canals	Super-resolution: CBCT → Micro CT
Sfeir et al. [88], Sfeir et al. [89] (2017, 2020)	CBCT	Linear model	First, premolar, first molar, second molar, incisor	CBCT Super-resolution
Ji et al. [90] (2024)	CBCT	Basicvsr++	First, molar	Super-resolution: CBCT → Micro CT
Zhang et al. [91] (2022)	X-ray	-	Second, molar root canals	-

**Table 6:** Overview of computational methods appearing in this review, grouped by primary task.

Task	Methods and Description
Image Classification/Prognosis Prediction	ResNet, VGG, Inception, CNNs, SVM, KNN, Random Forest (RF), Gradient Boosting Machine (GBM), Logistic Regression, Bayes Used to classify images, predict treatment options, or assess prognosis.
Segmentation of anatomical structures	U-Net (2D/3D), nnU-Net Automatically identifies and extracts root canal or filling regions from images.
Object Detection	YOLO network Detects regions of interest or pathological features on X-ray images.
Visualization/3D Reconstruction	3D Slicer, OsiriX MD, Materialise MiniMagics Used for visualizing and measuring root canal morphology and pulp volume.
